# Retinoic acid promotes second heart field addition and regulates ventral aorta patterning in zebrafish

**DOI:** 10.1016/j.ydbio.2025.03.013

**Published:** 2025-03-25

**Authors:** Austin H.C. Griffin, Allison M. Small, Riley D. Johnson, Anna M. Medina, Kiki T. Kollar, Ridha A. Nazir, Acasia M. McGuire, Jennifer A. Schumacher

**Affiliations:** a Department of Biology, Miami University, Oxford, OH, 45056, USA; b Department of Biological Sciences, Miami University, Hamilton, OH, 45011, USA

**Keywords:** Zebrafish, Retinoic acid, Heart development, Second heart field, Outflow tract, Aorta

## Abstract

Retinoic acid (RA) signaling is used reiteratively during vertebrate heart development. Its earliest known role is to restrict formation of the earlier-differentiating first heart field (FHF) progenitors, while promoting the differentiation of second heart field (SHF) progenitors that give rise to the arterial pole of the ventricle and outflow tract (OFT). However, requirements for RA signaling at later stages of cardiogenesis remain poorly understood. Here, we investigated the role of RA signaling after the later differentiating SHF cells have begun to add to the OFT. We found that inhibiting RA production in zebrafish beginning at 26 hours post fertilization (hpf) produced embryos that have smaller ventricles with fewer ventricular cardiomyocytes, and reduced number of smooth muscle cells in the bulbus arteriosus (BA) of the OFT. Our results suggest that the deficiency of the ventricular cardiomyocytes is due to reduced SHF addition to the arterial pole. In contrast to smaller ventricles and BA, later RA deficiency also results in a dramatically elongated posterior branch of the adjacent ventral aorta, which is surrounded by an increased number of smooth muscle cells. Altogether, our results reveal that RA signaling is required during the period of SHF addition to promote addition of ventricular cardiomyocytes, partition smooth muscle cells onto the BA and posterior ventral aorta, and to establish proper ventral aorta anterior-posterior patterning.

## 1. Introduction

The vertebrate heart is progressively built from two primary cell pools that are genetically and temporally distinct (Zaffran and Kelly, 2012). Earlier-differentiating first heart field (FHF) progenitors contribute to both the atrium and ventricle in zebrafish, which have a two-chambered heart, and to the left ventricle in mammals and birds, which have a four-chambered heart. In zebrafish, the later-differentiating second heart field (SHF) contributes cells to the arterial pole of the ventricle, the bulbus arteriosus (BA) of the outflow tract (OFT), and a small portion of the atrium (de Pater et al., 2009; Liu and Stainier, 2012). In animals with a four-chambered heart, the SHF contributes cells to both atria, the right ventricle, and the OFT (Buckingham et al., 2005). In zebrafish, the FHF contributes to the linear heart tube heart prior to 24 hours post fertilization (hpf), and the SHF adds to the poles of the heart after 24 hpf (de Pater et al., 2009). Hedgehog and FGF signaling pathways, as well as *mef2c* and *tbx1* transcription factors, play conserved roles to promote SHF addition to the arterial pole of the ventricle and the OFT (de Pater et al., 2009; Greulich et al., 2011; Hami et al., 2011; Hinits et al., 2012; Lazic and Scott, 2011; Lin et al., 1997; Nevis et al., 2013; Rochais et al., 2009). Yet, the molecular regulation of SHF addition remains incompletely understood.

Retinoic acid (RA), a derivative of vitamin A, plays a key role in vertebrate heart development. Increasing or decreasing RA signaling results in congenital heart defects (CHD) in vertebrates (D’Aniello and Waxman, 2015; Perl and Waxman, 2019; Stefanovic and Zaffran, 2017), indicating a highly conserved role for RA in heart development. Maternal vitamin A deficiency, which affects 15% of pregnant women (W.H.O., 2009), can lead to a variety of severe CHD in animal models, with similar defects seen in humans (Dersch and Zile, 1993; Wilson et al., 1953; Wilson and Warkany, 1950a, 1950b). The earliest known role of RA is to restrict the size of the cardiomyocyte progenitor population formed in the anterior lateral plate mesoderm (Collop et al., 2006; Keegan et al., 2005; Sirbu et al., 2008; Waxman et al., 2008). Mice lacking the RA-synthesizing enzyme retinaldehyde dehydrogenase-2 (Raldh2) have cardiomegaly, with a posterior expansion of the cardiac progenitor field (Niederreither et al., 1999; Ryckebusch et al., 2008; Sirbu et al., 2008). Inhibiting RA signaling in zebrafish prior to or during gastrulation using the retinaldehyde dehydrogenase inhibitor diethyl-aminobenzoic acid (DEAB), which prevents RA production, resulted in larger hearts with increased number of cardiomyocytes (Keegan et al., 2005; Waxman et al., 2008). More recently it was shown that early inhibition of RA specifically resulted in larger hearts with increased number of FHF-derived cardiomyocytes, but a dramatic reduction in the differentiation of SHF-derived cells contributing to the ventricle (Duong et al., 2021). These results indicated that RA plays a role in SHF cell maintenance prior to 24 hpf, potentially through inhibiting their premature differentiation. Application of exogenous RA after 24 hpf resulted in smaller hearts, with fewer SHF cardiomyocytes in the ventricle (Rydeen and Waxman, 2016), pointing to a potential later role for RA signaling during the time of SHF addition. However, studies examining the role of endogenous RA in ventricle and OFT development specifically during the time period of SHF addition are fairly limited. Knockout of the RA receptors RARα1 and RXRα in mice starting at the heart tube stage, when SHF addition begins, led to defects in the OFT and the adjacent great vessels that are consistent with reduced SHF contribution (Li et al., 2010). The same study found that RARα1/RARβ1 double mutants had a specific loss of the SHF cells in the splanchnic mesoderm flanking the OFT starting at the heart looping stage, which is approximately 24 hours after SHF addition begins. Notably, this study made use of RA receptor knockout strategies, and it is possible that residual RA signaling remained in these embryos via the use of alternative receptors, which would give an incomplete view of the full role for RA. Another study found that administration of an RA antagonist to pregnant mice during the early part of SHF addition (E7.75 and E8.25) did not lead to any overt defects in the right ventricle as examined at E15.5 (De Bono et al., 2018). However, the chemical inhibition strategy that was used did not inhibit RA signaling during the entire period of SHF addition, which extends through E10.5 (Kelly et al., 2001). Neither study quantified right ventricular cardiomyocyte number or SHF addition, and given the limitations of both studies, there are still open questions regarding the role of endogenous RA signaling during the time of SHF addition. No zebrafish studies to date have specifically examined the role of endogenous RA signaling in ventricle and OFT development after 24 hpf, during the time of SHF addition.

In animals with four-chambered hearts, the aorta carries blood from the left ventricle into systemic circulation. In mice, RA plays an important role in aortic arch formation via its roles in SHF development, cardiac neural crest distribution, and patterning the pharyngeal arch arteries (PAAs), which will remodel to form the aortic arch and its major arterial branches (Anderson and Bamforth, 2022; Hiruma et al., 2002). The PAAs and their associated pharyngeal endoderm pouches form in a rostral to caudal sequence, and RA is primarily involved in patterning the caudal PAAs and pouches (Kopinke et al., 2006; Li et al., 2012; Niederreither et al., 2003; Vermot et al., 2003). RA signaling is known to regulate cardiac neural crest contribution and function within the OFT, which is important for OFT septation and cushion development (El Robrini et al., 2016; Jiang et al., 2002). Reduction of RA signaling leads to a variety of great vessel defects including transposition of the great arteries, persistent truncus arteriosus, interrupted aortic arch, as well as benign variants of aortic arch structure (Cipollone et al., 2006; El Robrini et al., 2016; Kastner et al., 1994; Lee et al., 1997; Mendelsohn et al., 1994; Sugrue et al., 2019; Sugrue and Zohn, 2019). In the zebrafish two-chambered heart, the ventral aorta is the sole vessel receiving blood from the single ventricle. Compared to mouse models, very little is known regarding the cellular and molecular mechanisms driving zebrafish ventral aorta formation. The ventral aorta is initially bilateral at 48 hpf, but by 72 hpf the vessels have rearranged into a singular midline vessel (Anderson et al., 2008). The majority of the ventral aorta extends anteriorly from the BA (Isogai et al., 2001). However, there is a very small segment of the ventral aorta that extends posteriorly from the BA, and ends at PAA six (Anderson et al., 2008). As in mouse, RA is required for proper formation of the caudal PAAs and associated endodermal pouches in zebrafish (Begemann et al., 2001; Kopinke et al., 2006; Li et al., 2012; Linville et al., 2009), opening the possibility of a conserved role for RA in zebrafish ventral aorta patterning.

Both the ventral aorta and the BA are covered by a continuous sheath of smooth muscle cells. The smooth muscle of the BA is derived from the SHF and cardiac neural crest (Cavanaugh et al., 2015; Hami et al., 2011). Mesenchymal cells that will contribute to the smooth muscle of the ventral aorta first appear by 58 hpf, and they transition to a smooth muscle appearance starting at 66 hpf (Whitesell et al., 2019). By 4 dpf, the ventral aorta is surrounded by a single layer of smooth muscle cells (Whitesell et al., 2014). Prior studies investigating the mechanisms driving smooth muscle coverage of the ventral aorta were limited to smooth muscle cells in region of the ventral aorta that extends anteriorly from the BA, which are primarily neural crest in origin (Cavanaugh et al., 2015). To our knowledge, no studies have examined coverage of the posterior region of the ventral aorta. Additionally, the role of RA signaling in ventral aorta smooth muscle recruitment has not been investigated.

Here, we assessed the requirement of RA signaling in the zebrafish heart after 24 hpf, when SHF progenitors have begun to add to the poles of the heart. We found that embryos with later RA deficiency had linear hearts with fewer ventricular cardiomyocytes and fewer smooth muscle cells within the BA at 72 hpf, due at least in part to reduced addition of SHF cells. We also found that this comparatively later RA deficiency produced a posterior expansion of the smooth muscle cells, which typically surround only the BA of the OFT at 72 hpf, to also cover the ventral aorta. Furthermore, the expansion of smooth muscle cells onto the posterior branch of the ventral aorta occurred in conjunction with an expansion of the posterior segment itself and a reduction in the anterior segment, consistent with a role of RA signaling in anterior-posterior patterning of the ventral aorta. Our results also reveal that RA signaling regulates remodeling of BA-ventral aorta junction position. Together, our results reveal previously unrecognized roles for RA signaling in promoting SHF addition to the arterial pole of the heart and in anterior-posterior patterning of the ventral aorta.

## 2. Results

### 2.1. Embryos with RA deficiency after 24 hpf have smaller ventricles

**Fig. 1.**
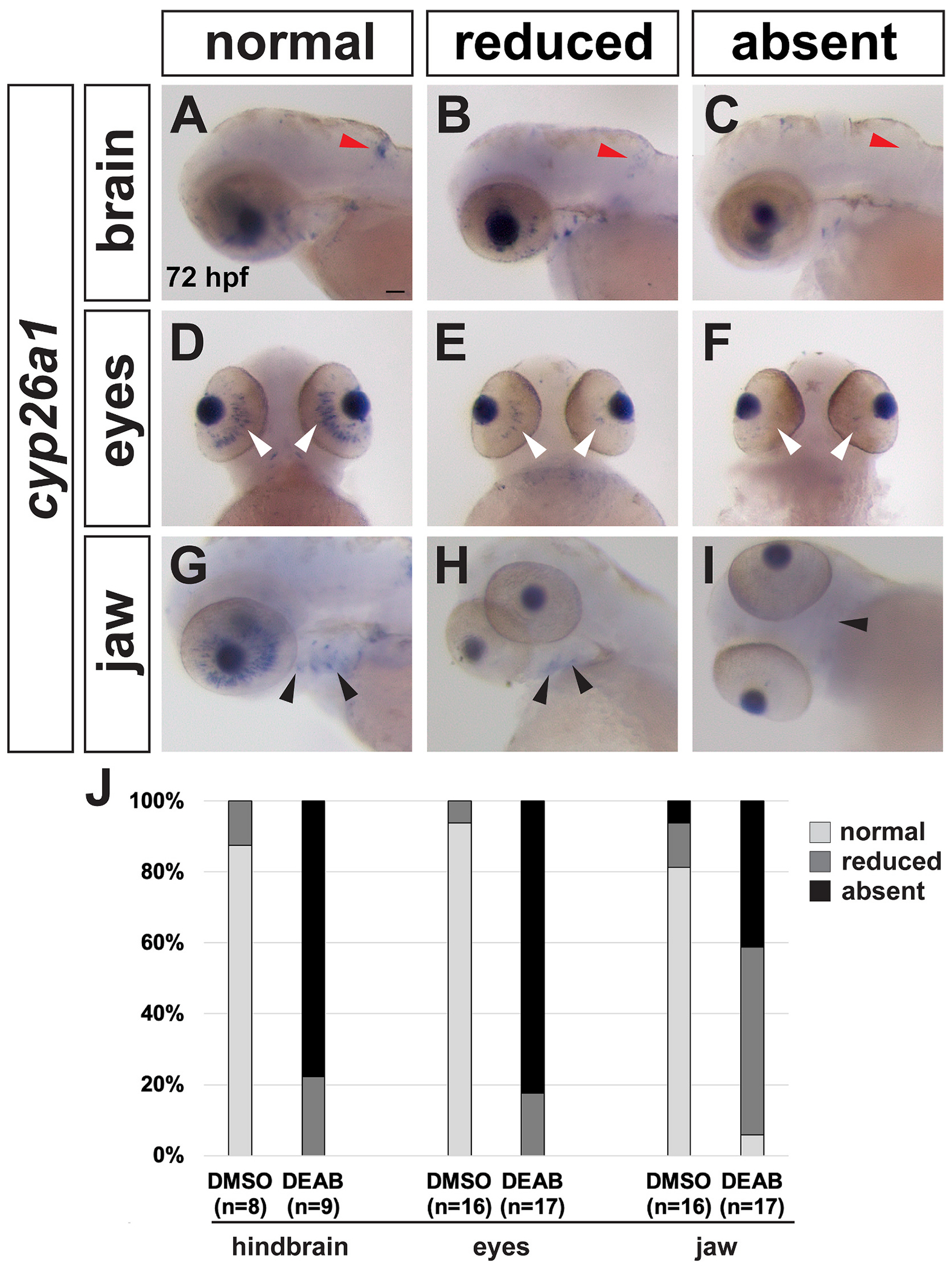
*Cyp26a1* expression is reduced in embryos treated with DEAB from 26 hpf to 72 hpf. *cyp26a1* expression in the hindbrain (A-C, red arrowheads), eyes (D-F, white arrowheads) and jaw (G-I, black arrowheads) is strongly reduced or absent in DEAB-treated embryos. J shows quantification of expression in each domain. All embryos are 72 hpf. Scale bar is 50 μM. (For interpretation of the references to colour in this figure legend, the reader is referred to the Web version of this article.)

**Fig. 2.**
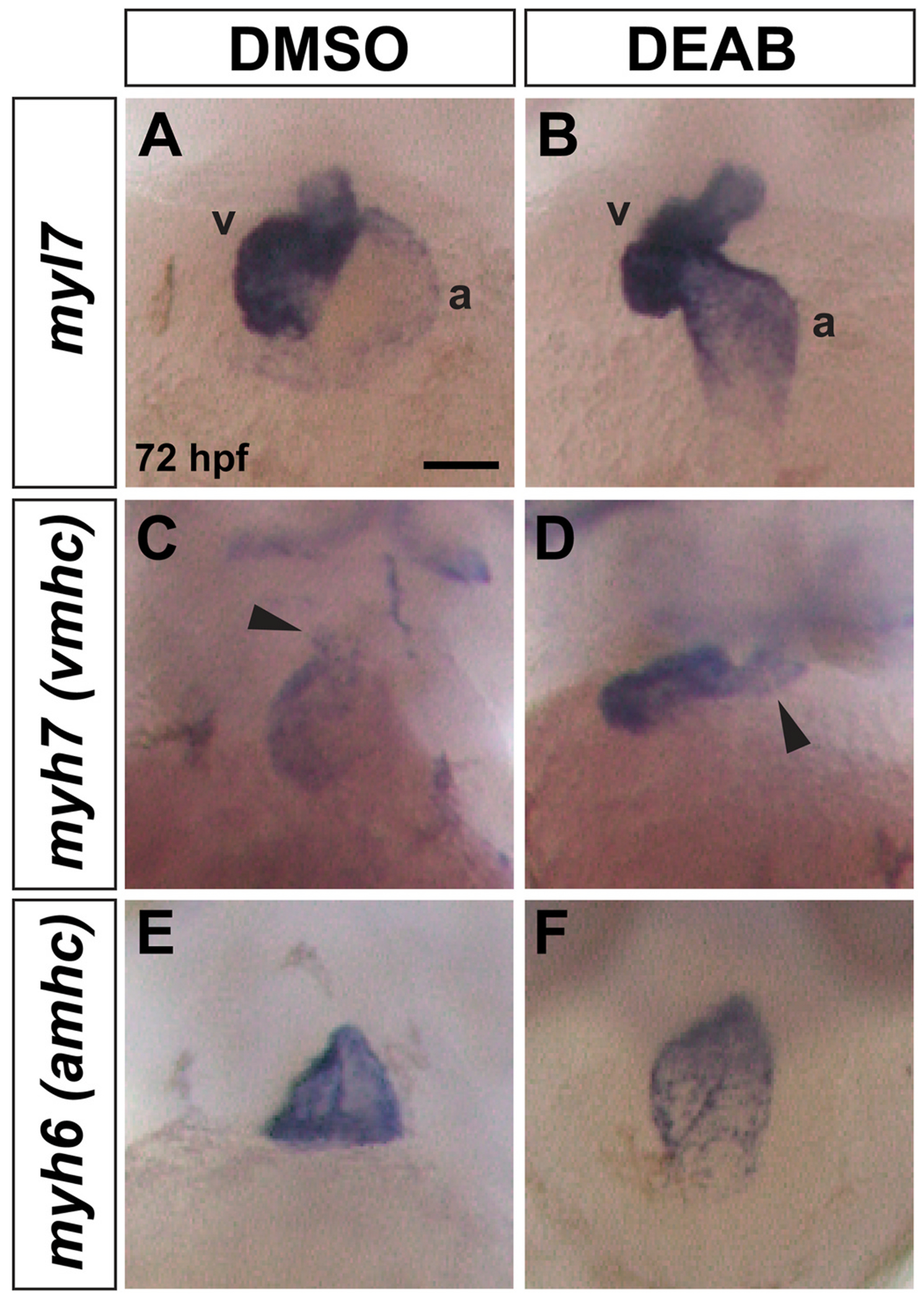
Heart morphology is abnormal in late RA-deficient embryos. (A, B) *myl7* expression is similar in the ventricle (v) and atrium (a) of control (n = 35/35 have normal morphology as shown) and DEAB-treated embryos (n = 27/37 have elongated, linear morphology as shown, 10/37 have more horizontal chamber placement as seen in controls). (C, D) *myh7* expression is present in the ventricle of DEAB-treated embryos (n = 31), but the ventricle is more compact with an elongated OFT region compared to the controls (n = 23). For control embryos, 21/23 have short OFT as shown, 2/23 have elongated OFT. For DEAB-treated embryos, 10/31 have short OFT, 21/31 have elongated OFT as shown. (E, F) *myh6* expression is present in the atrium of DEAB-treated embryos but the chamber morphology is abnormal compared to controls. For control embryos, 48/48 have normal morphology as shown. For DEAB-treated embryos, 32/39 have variable abnormal elongated morphology. All embryos are 72 hpf. Scale bar is 50 μM.

To investigate if RA signaling is required during SHF addition, we treated embryos with DMSO (control) or the retinaldehyde dehydrogenase inhibitor DEAB (to block RA production) starting at 26 hpf, just after SHF cells begin to differentiate, which we refer to as late RA inhibition or deficiency. We confirmed effective inhibition of RA signaling in DEAB-treated embryos by examining expression of the RA target gene *cyp26a1* (Loudig et al., 2000) using in situ hybridization (ISH). All *cyp26a1* expression domains, including the hindbrain (Fig. 1A–C), eye (Fig. 1D–F), and pharyngeal region (Fig. 1G–I), were strongly reduced or absent following late RA inhibition (Fig. 1J). Given that we were able to deplete RA signaling at these later stages of cardiogenesis, we then examined heart morphology at 72 hpf by ISH with the pan-cardiac marker *myl* (Fig. 2A and B) (Yelon et al., 1999), the ventricle-specific marker *vmhc* (Fig. 2C and D)*,* and the atrial-specific marker *amhc* (Fig. 2E and F). Expression of all three markers appeared normal, with *vmhc* and *amhc* appropriately restricted to the ventricle and atrium, respectively. However, hearts in late RA-deficient embryos were elongated with more compact chambers compared to the DMSO controls (Fig. 2A and B). Specifically, the ventricle appeared more compact and smaller, with an elongated OFT region, in late RA-deficient embryos compared to controls (Fig. 2C and D), suggesting that ventricle growth might be affected in late RA-deficient embryos.

**Fig. 3.**
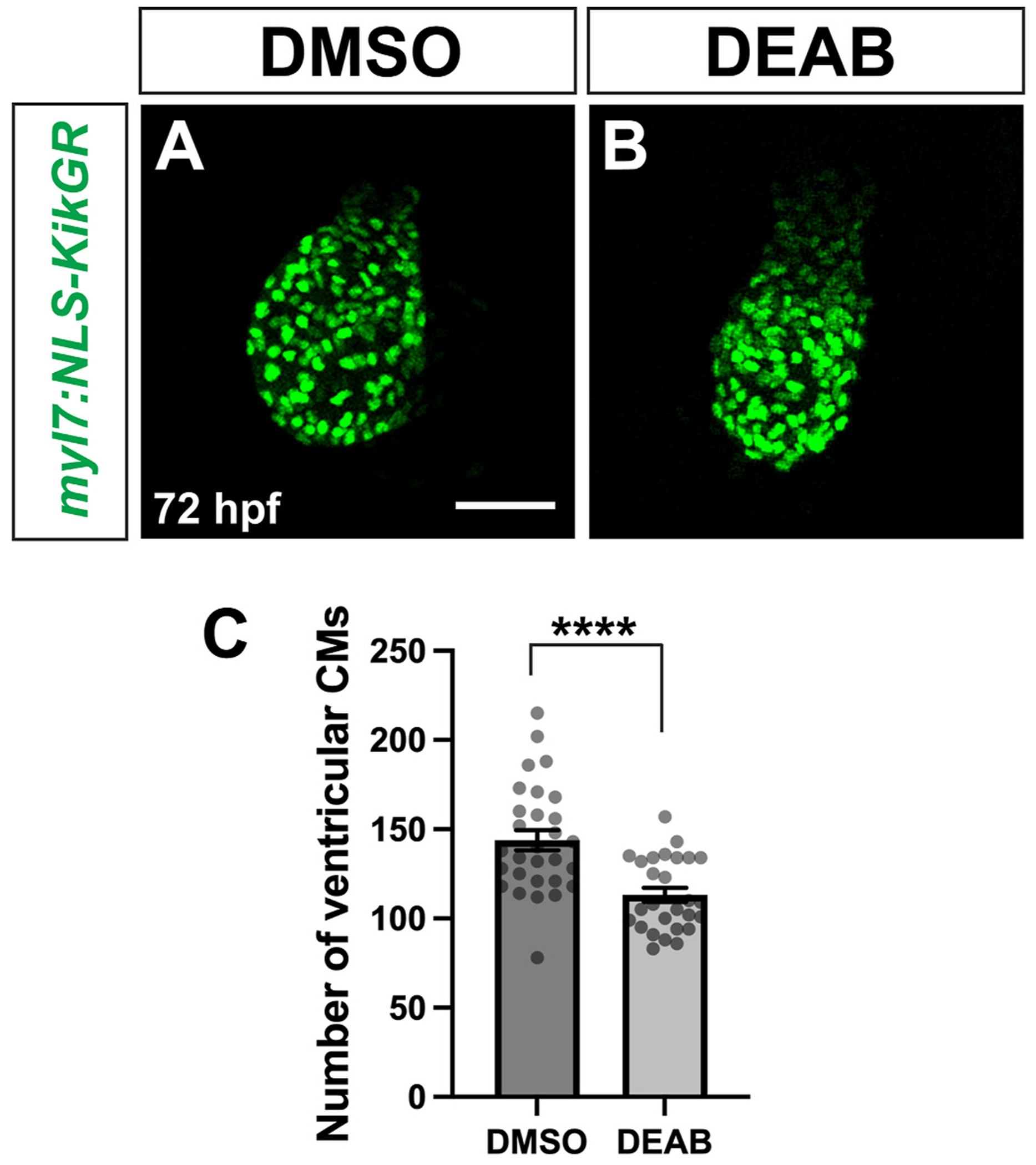
Ventricular cardiomyocyte number is reduced in late RA-deficient embryos. (A, B) Representative images of *my7:NLS-KikGR* expression in the ventricle of control DMSO (A) and DEAB-treated (B) embryos at 72 hpf. Note that expression is absent in the atrium, preventing analysis of that chamber. Maximum intensity projections of a Z-stack are shown for each image. Scale bar is 50 μM. (C) Number of ventricular cardiomyocytes at 72 hpf in control (n = 29) and DEAB-treated (n = 27) embryos. Error bars represent SEM. **** indicates p < 0.0001.

**Fig. 4.**
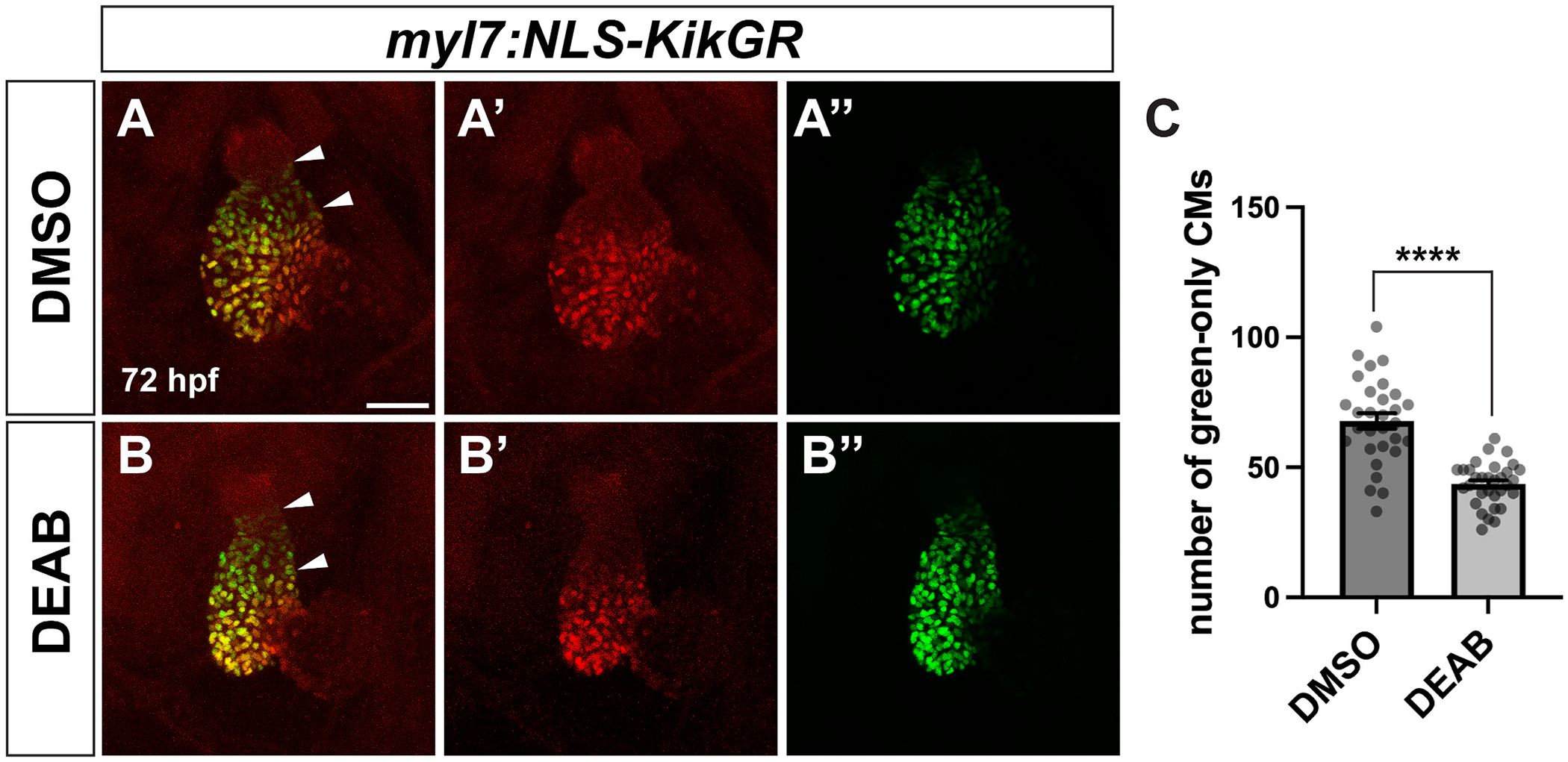
SHF addition to the arterial pole of the ventricle is reduced in late RA-deficient embryos. (A, B) Representative images of temporal differentiation assay using the *myl7:NLS-KikGR* transgene in control DMSO (A) and DEAB-treated (B) embryos. White arrowheads indicate region of green-only later-differentiating cardiomyocytes of the SHF at the arterial pole. (A′, B′) Red channel only. (A″, B″) Green channel only. Maximum intensity projections of a Z-stack are shown for each image. Scale bar is 50 μM. (C) Quantification of green-only cardiomyocytes at 72 hpf in control (n = 30) and DEAB-treated (n = 32) embryos. Error bars represent SEM. **** indicates p < 0.0001. (For interpretation of the references to colour in this figure legend, the reader is referred to the Web version of this article.)

In zebrafish, the ventricle increases in cell number from 24 hpf to 72 hpf, primarily via addition of SHF cells to the arterial pole (de Pater et al., 2009; Lazic and Scott, 2011), which correlated with the timing of our DEAB treatments. To investigate if the compact ventricle in late RA-deficient embryos was due to changes in cardiomyocyte number, we quantified the number of ventricular cardiomyocytes at 72 hpf using the *myl7:NLS-KikGR* transgenic line (Lazic and Scott, 2011). Although the *myl7* promoter should drive nuclear KikGR expression in both the ventricle and the atrium, we found that NLS-KikGR fluorescence in the atrium was too dim to accurately quantify cardiomyocytes. Therefore, we focused on the ventricle. We found that embryos with late RA-deficiency had significantly fewer ventricular cardiomyocytes than controls (Fig. 3A–C). Previous studies have shown that a reduction in ventricular cardiomyocyte number can arise from defects in addition of SHF cells to the arterial pole of the heart (Holowiecki et al., 2020; Lazic and Scott, 2011; Rydeen and Waxman, 2016; Song et al., 2019). To quantify SHF addition, we performed a temporal differentiation assay (Rydeen and Waxman, 2016). Embryos carrying the *myl7:NLS-KikGR* transgene were photoconverted from green-to-red at 26 hpf, which results in all *myl7:NLS-KikGR*+ cardiomyocytes, which are primarily FHF-derived cardiomyocytes, being marked in red. Immediately following the photoconversion, embryos were treated with DEAB through 72 hpf, as above. We found that by 72 hpf the number of ventricular cardiomyocytes that were later-differentiating progeny of the SHF, which are the cells that added to the heart after 26 hpf and appeared only green, were significantly reduced compared to green-only ventricular cardiomyocytes in control embryos (Fig. 4A–C). Therefore, our data are consistent with the smaller, more compact ventricles in embryos with later RA deficiency being due at least in part to reduced SHF addition.

**Fig. 5.**
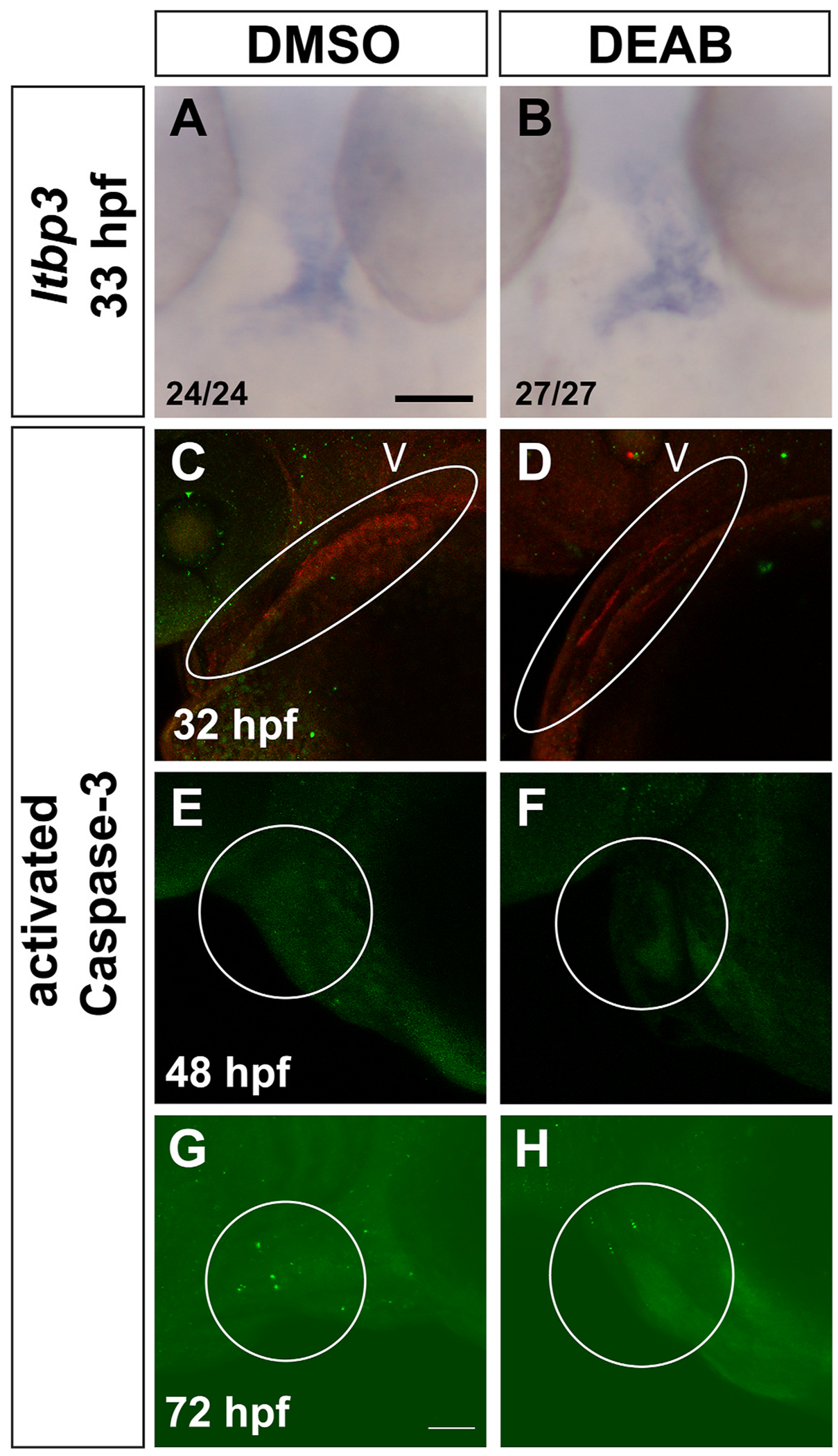
The SHF progenitor pool is present, and apoptosis is not increased in late RA-deficient embryos. (A–B) *ltbp3* expression in embryos treated with DMSO or DEAB from 26 hpf to 33 hpf. (C, D) Activated Caspase-3 distribution (green) at 32 hpf in the heart (indicated by MF20 staining in red) and adjacent regions in embryos treated with DMSO (n = 26/28 with no staining in heart) or DEAB (n = 27/29 with no staining in heart). White ovals outline the heart region, V indicates ventricle region of heart tube. (E, F) Activated Caspase-3 distribution at 48 hpf in the heart and adjacent regions in embryos treated with DMSO (n = 21/21 with no staining in the heart) or DEAB (n = 16/18 with no staining in the heart). (G, H) Activated Caspase-3 distribution at 72 hpf in the heart and adjacent regions in embryos treated with DMSO (n = 31/32 as shown) or DEAB (n = 31/32 as shown). White circles outline the ventricle and OFT region in E-H. Maximum intensity projections of a Z-stack are shown for each image. Anterior is to the left. Scale bars are 50 μM. (For interpretation of the references to colour in this figure legend, the reader is referred to the Web version of this article.)

**Fig. 6.**
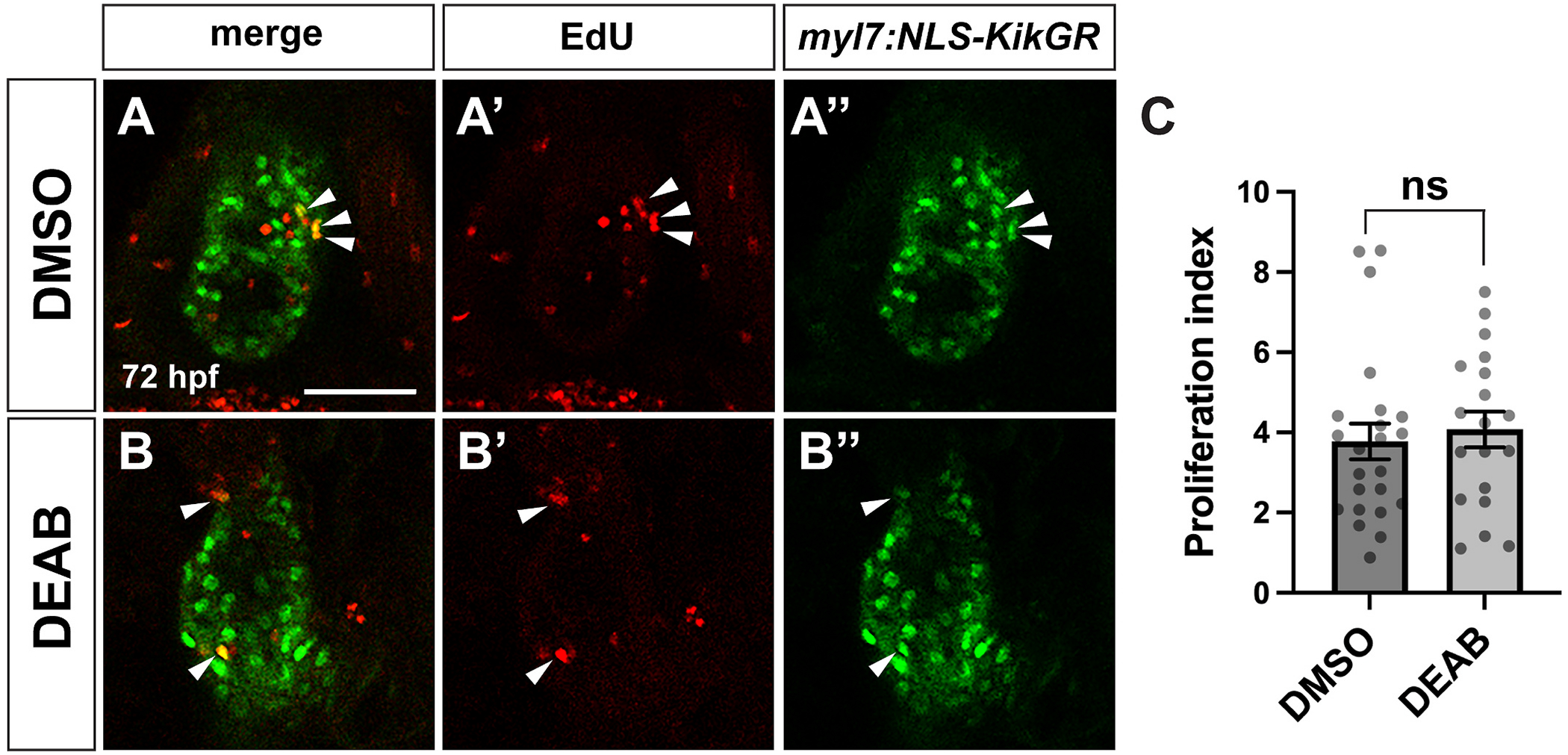
Ventricular cardiomyocyte proliferation is unchanged in late RA-deficient embryos (A, B) Representative images of EdU staining in control DMSO (A) and DEAB-treated (B) embryos at 72 hpf. (A′, B′) EdU in red and (A″, B″) *myl7:NLS-KikGR* transgene expression in cardiomyocytes shown in green. Arrowheads indicate EdU+/NLS-KikGR + cardiomyocytes. Single confocal sections are shown. Scale bar is 50 μM. (C) Proliferation indices for DMSO (n = 23) and DEAB-treated (n = 19) embryos. (For interpretation of the references to colour in this figure legend, the reader is referred to the Web version of this article.)

The SHF progenitor pool is established in the ALPM prior to 24 hpf (Guner-Ataman et al., 2013; Lazic and Scott, 2011), so our DEAB treatments at 26 hpf should not affect the initial establishment of the pool. However, reduced SHF addition could be due to a later reduction in the SHF progenitors. To determine if the SHF progenitors were reduced or absent following RA inhibition, we examined *ltbp3*, which is expressed in the SHF adjacent to the ventricle by 30 hpf (Zhou et al., 2011). The overall pattern of staining, including both the size and shape of the *ltbp3* expression domain, was overtly normal in late RA-deficient embryos compared to controls (Fig. 5A and B). Therefore, the SHF progenitor pool is still present, at least for several hours following late RA inhibition. Alternatively, SHF cells that are added to the arterial pole in late RA-deficient embryos could undergo apoptosis later in development, leading to the reduced ventricular cell number. To test for this, we examined activated Caspase-3 distribution at 32, 48, and 72 hpf in late RA-deficient embryos. However, we found no significant increases in apoptosis in the region of the ventricle and arterial pole at any stage (Fig. 5C–H). Therefore, it is unlikely that apoptotic cell death contributes to the reduced number of ventricular cardiomyocytes or SHF cells. An additional possibility is that the smaller ventricles in late RA-deficient embryos could be due to reduced cardiomyocyte proliferation. To test for this, we pulsed embryos carrying the *myl7:NLS-KikGR* transgene with EdU at 26 hpf to label proliferating cells, then immediately treated with DMSO or DEAB until 72 hpf as in our other experiments. Using this strategy, there was not a significant change in the proliferation index in late RA-deficient embryos compared to controls (Fig. 6A–C).

### 2.2. Late RA-deficient embryos have abnormal distribution of smooth muscle cells in the outflow tract

**Fig. 7.**
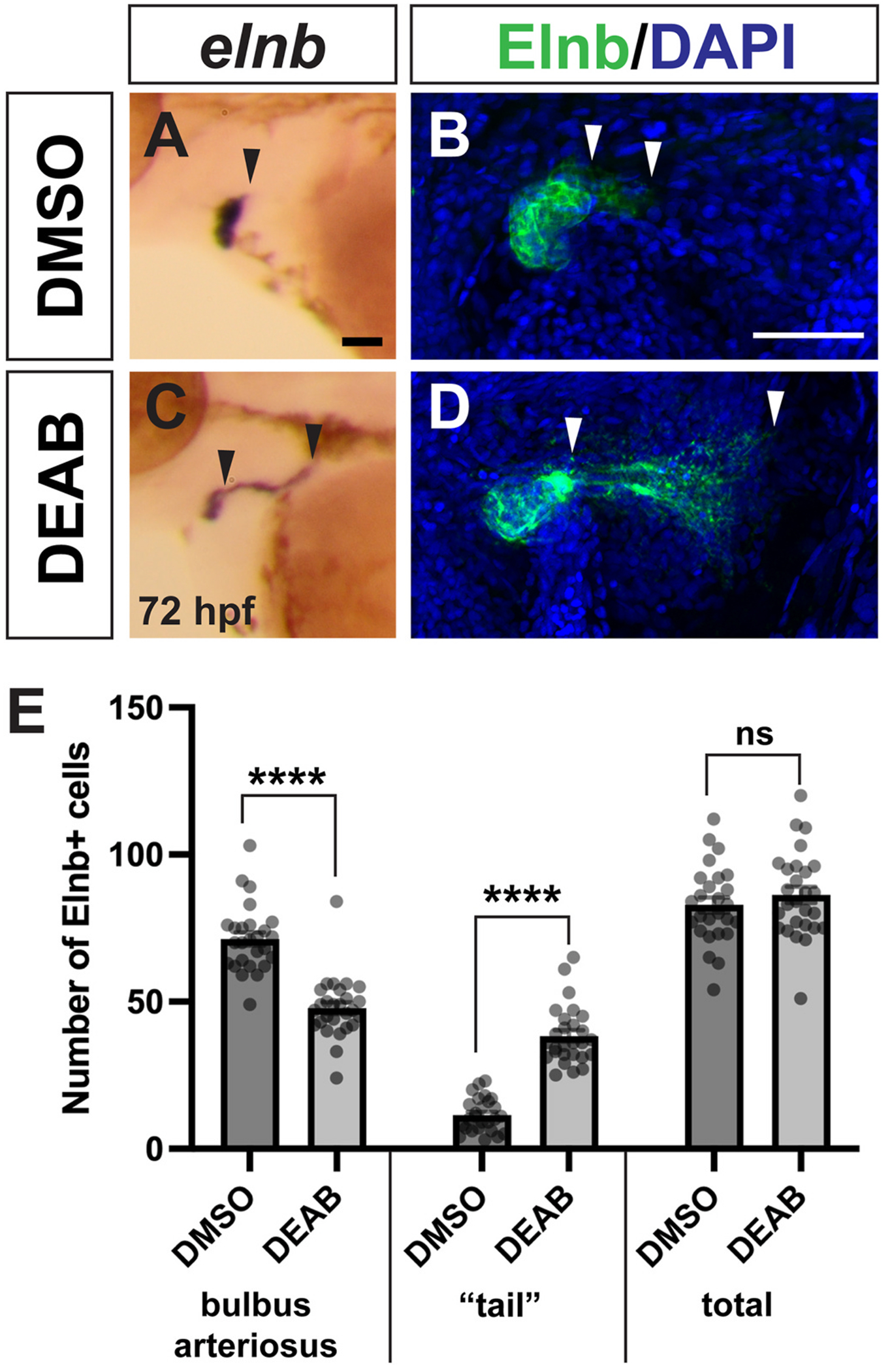
OFT smooth muscle distribution is altered in late RA-deficient embryos. (A, B) ISH for *elnb* expression in the OFT in control (n = 12) and DEAB-treated (n = 19) embryos at 72 hpf. Black arrowheads indicate posterior *elnb* expression domain. (C, D) Representative images of immunostaining for Elnb protein in the OFT in control and DEAB-treated embryos at 72 hpf. Maximum intensity projections are shown. DAPI marks all nuclei in blue, Elnb expression is shown in green. White arrowheads indicate boundaries of the “tail”, or posterior Elnb expression domain. Maximum intensity projections of a Z-stack are shown for each image. (E) Quantification of Elnb expression in smooth muscle cells of the BA, “tail” region, and total cell number in DMSO (n = 26) and DEAB-treated (n = 27) embryos. Scale bars are 50 μM, anterior to the left in all images. Error bars represent SEM. **** indicates p < 0.0001, ns = not significant. (For interpretation of the references to colour in this figure legend, the reader is referred to the Web version of this article.)

SHF cells also contribute to smooth muscle of the bulbus arteriosus (BA), which is adjacent to the arterial pole of the ventricle. We examined BA morphology using ISH for *elnb*, which is expressed exclusively in the smooth muscle of the BA at 72 hpf (Miao et al., 2007). Surprisingly, we found a dramatic expansion of the *elnb* expression domain in late RA-deficient embryos (Fig. 7A and C). To examine this defect at higher resolution, we performed immunohistochemistry for Elnb. Whereas DMSO control embryos had a cap of smooth muscle cells surrounding the BA and a very short posterior “tail” of Elnb expression, the “tail” domain was expanded posteriorly in late RA-deficient embryos, and the cap surrounding the BA appeared reduced (Fig. 7B and D). We further quantified Elnb expression by counting the number of Elnb-expressing cells in the domains surrounding the BA and the posterior “tail” domain. While the total number of Elnb-expressing smooth muscle cells was unchanged in late RA-deficient embryos, we found that the cell distribution was dramatically altered. There were significantly fewer smooth muscle cells surrounding the BA, and significantly more smooth muscle cells in the posterior “tail” domain (Fig. 7E).

**Fig. 8.**
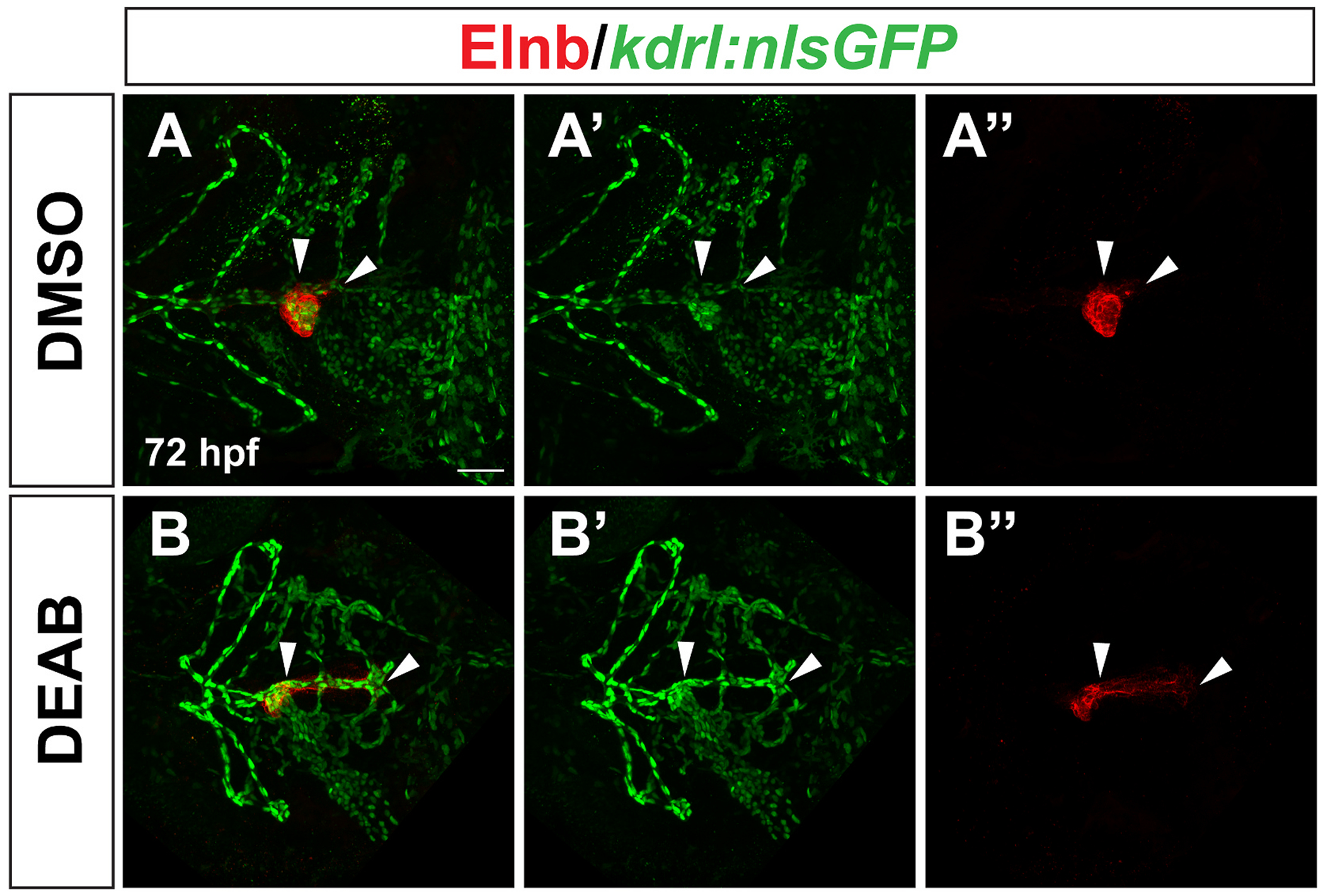
The expanded posterior smooth muscle domain in late RA-deficient embryos surrounds an elongated posterior branch of the ventral aorta. Representative images of *kdrl:nlsGFP* expression detected by immunostaining in endothelial cells (A′, B′), Elnb expression in OFT smooth muscle (A″, B″), and the merged images (A, B). n = 16 for DMSO control, n = 24 for DEAB. White arrowheads indicate the posterior region of the ventral aorta in A′ and B′, the posterior “tail” of Elnb expression in A″ and B″, and the overlap of expression where the posterior Elnb domain surrounds the posterior region of the ventral aorta in A and B. Maximum intensity projections of a Z-stack are shown for each image. Scale bar is 50 μM, anterior is to the left in all images.

Starting at 58 hpf, smooth muscle cells begin to attach to the ventral aorta, which extends anteriorly from the OFT (Whitesell et al., 2019). However, the smooth muscle expansion we observed in late RA-deficient embryos extended posteriorly. To determine if this posteriorly-expanded smooth muscle domain surrounded a blood vessel, we examined Elnb expression in embryos carrying the *kdrl:nlsGFP* transgene, which is expressed in vascular endothelial cells (Blum et al., 2008). In control embryos, the small posterior region of Elnb expression surrounded a very short segment of the ventral aorta extending posterior to the BA (Fig. 8A). However, in late RA-deficient embryos, the Elnb-expressing smooth muscle surrounded a dramatically elongated posterior segment of the ventral aorta (Fig. 8B).

### 2.3. Late RA deficiency leads to mis-patterning of the ventral aorta and abnormal position of the junction between the outflow tract and ventral aorta

**Fig. 9.**
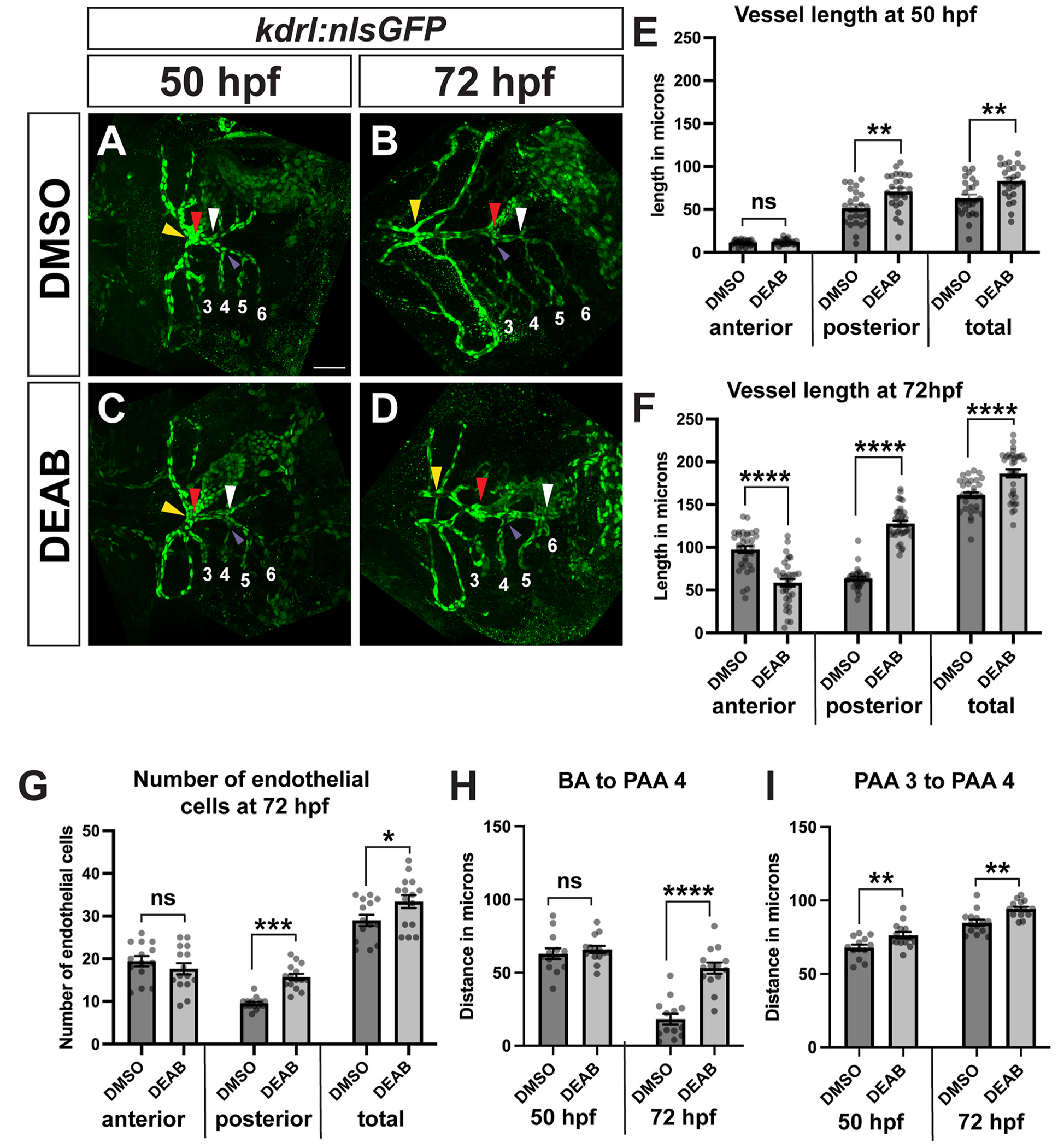
The posterior ventral aorta domain is expanded in late RA-deficient embryos. (A–D) Representative images of immunostaining to detect *kdrl:nlsGFP* expression in endothelial cells at 50 hpf (A, C) and 72 hpf (B, D) in control and DEAB-treated embryos. Red arrowheads indicate center of the BA, which represents the boundary between anterior and posterior segments, yellow arrowheads indicate anterior end of fused ventral aorta, white arrowheads indicate posterior end of ventral aorta. Numbers indicate PAA positions. Purple arrowheads indicate the junction of PAA 4 and the ventral aorta. For all analyses, the distance between the yellow arrowhead and the red arrowhead represents the length of the anterior segment, and the distance between the white arrowhead and the red arrowhead represents the length of the posterior segment. (E) Quantification of ventral aorta lengths at 50 hpf in control (n = 25) and DEAB-treated (n = 25) embryos. (F) Quantification of ventral aorta lengths at 72 hpf in control (n = 33) and DEAB-treated (n = 34) embryos. (G) Quantification of distance between the OFT and PAA 4 at 50 hpf in control (n = 13) and DEAB-treated (n = 14) embryos and 72 hpf in control (n = 14) and DEAB-treated (n = 14) embryos. (H) Quantification of distance between PAA 3 and PAA 4 at 50 hpf in control (n = 13) and DEAB-treated (n = 14) embryos and 72 hpf in control (n = 14) and DEAB-treated (n = 14) embryos. (I) Endothelial cell number in ventral aorta segments at 72 hpf in control (n = 14) and DEAB-treated (n = 15) embryos. Scale bar is 50 μM. Error bars represent SEM. **** indicates p < 0.0001, *** indicates p < 0.001, ** indicates p < 0.01, * indicates p < 0.05, ns = not significant. (For interpretation of the references to colour in this figure legend, the reader is referred to the Web version of this article.)

To better understand the etiology of the ventral aorta patterning defects in late RA-deficient embryos, we analyzed ventral aorta patterning using the *kdrl:nlsGFP* transgene. In control embryos at 50 hpf, both anterior and posterior ventral aorta segments were almost completely bilaterally split, with only the region immediately adjacent to the BA being fused at the midline (Fig. 9A). The length of the anterior fused segment of late RA-deficient embryos did not differ significantly from control embryos (Fig. 9C and E). However, in late RA-deficient embryos at 50 hpf, the posterior segment had a significantly longer fused region compared to control embryos (Fig. 9C and E). Furthermore, the total length of the fused ventral aorta was significantly longer in late RA-deficient embryos (Fig. 9E).

Because we initially observed the posteriorly-expanded smooth muscle and elongated posterior ventral aorta at 72 hpf, we also quantified the ventral aorta segment lengths at this later stage. In control embryos at 72 hpf, the anterior segment of the ventral aorta had undergone significant outgrowth and fusion (Fig. 9B), increasing dramatically in length (Fig. 9F). The posterior segment, which had fused to the position of PAA five, was much shorter than the anterior segment (Fig. 9B and F). In late RA-deficient embryos, the fused anterior ventral aorta segment was significantly shorter than controls (Fig. 9F) and gaps were often present in the vessel, with fusion occurring at a distance from the BA (Fig. 9D). The posterior fused segment measured significantly longer than control embryos (Fig. 9F). The total length of the fused ventral aorta at 72 hpf was also significantly increased in late RA-deficient embryos (Fig. 9F). Taken together, these results are consistent with a role for RA signaling in ventral aorta anterior-posterior patterning.

To determine a potential cause for the increased ventral aorta length, we counted the number of endothelial cells located in the fused portions in the anterior and posterior segments using the *kdrl:nlsGFP* transgene. The increase in the total number of endothelial cells in late RA-deficient embryos compared to controls was small but statistically significant (Fig. 9G), with a specific increase in the number of cells in the posterior segment. The anterior segment slightly trended towards a decrease in the number of cells, however this difference was not statistically significant (Fig. 9G). These results suggest that an increase in cell number contributes in part to the increase in overall length. However, given the dramatic length increase and more subtle cell number increase, there are likely other mechanisms driving the length increase, potentially via endothelial cell shape changes.

With the knowledge that RA plays an important role in PAA formation, we also examined the position of the PAAs, with particular attention to the relative positioning of the BA to the neighboring PAAs. We noticed that the connection between the BA and the ventral aorta appeared to shift in control embryos, with the BA positioned close to PAA 3 at 50 hpf (distance between the red and purple arrowheads in Fig. 9A), and by 72 hpf it was very close to PAA 4 (distance between the red and purple arrowheads in Fig. 9B). Because this rearrangement has not been reported previously, we sought to clearly quantify this change in position. We measured the distance from PAA 4 to the BA, as well as the distance between PAAs 3 and 4. In control embryos, at 50 hpf the BA was approximately 63 ± 4 μm anterior to PAA 4, and the distance between PAAs 3 and 4 was approximately 66 ± 2.4 μm, placing the BA very close to PAA 3 (Fig. 9H and I). By 72 hpf, the distance from the BA to PAA 4 had decreased to approximately 16 ± 4 μm, while the distance between PAAs 3 and 4 had increased to approximately 85 ± 8 μm (Fig. 9H and I). Therefore, our measurements in control embryos confirm that the BA position does rearrange, and that the distance between PAAs 3 and 4 increases between 50 hpf and 72 hpf. In late RA-deficient embryos, the BA was positioned normally at 50 hpf relative to PAA 4, however the overall distance between PAAs 3 and 4 was increased (Fig. 9H and I). By 72 hpf in late RA-deficient embryos, the distance from the BA to PAA 4 decreased only slightly, and was significantly greater than the distance in control embryos (Fig. 9H and I), indicating that the remodeling seen in control embryos did not occur properly. The distance between PAAs 3 and 4 was still increased compared to controls (Fig. 9H and I). Together, these results are consistent with a role for endogenous RA in coordinating the remodeling of the junction between the BA and the ventral aorta relative to the arch arteries.

### 2.4. RA acts between 26 hpf and 50 hpf to distribute smooth muscle cells of the outflow tract and ventral aorta

**Fig. 10.**
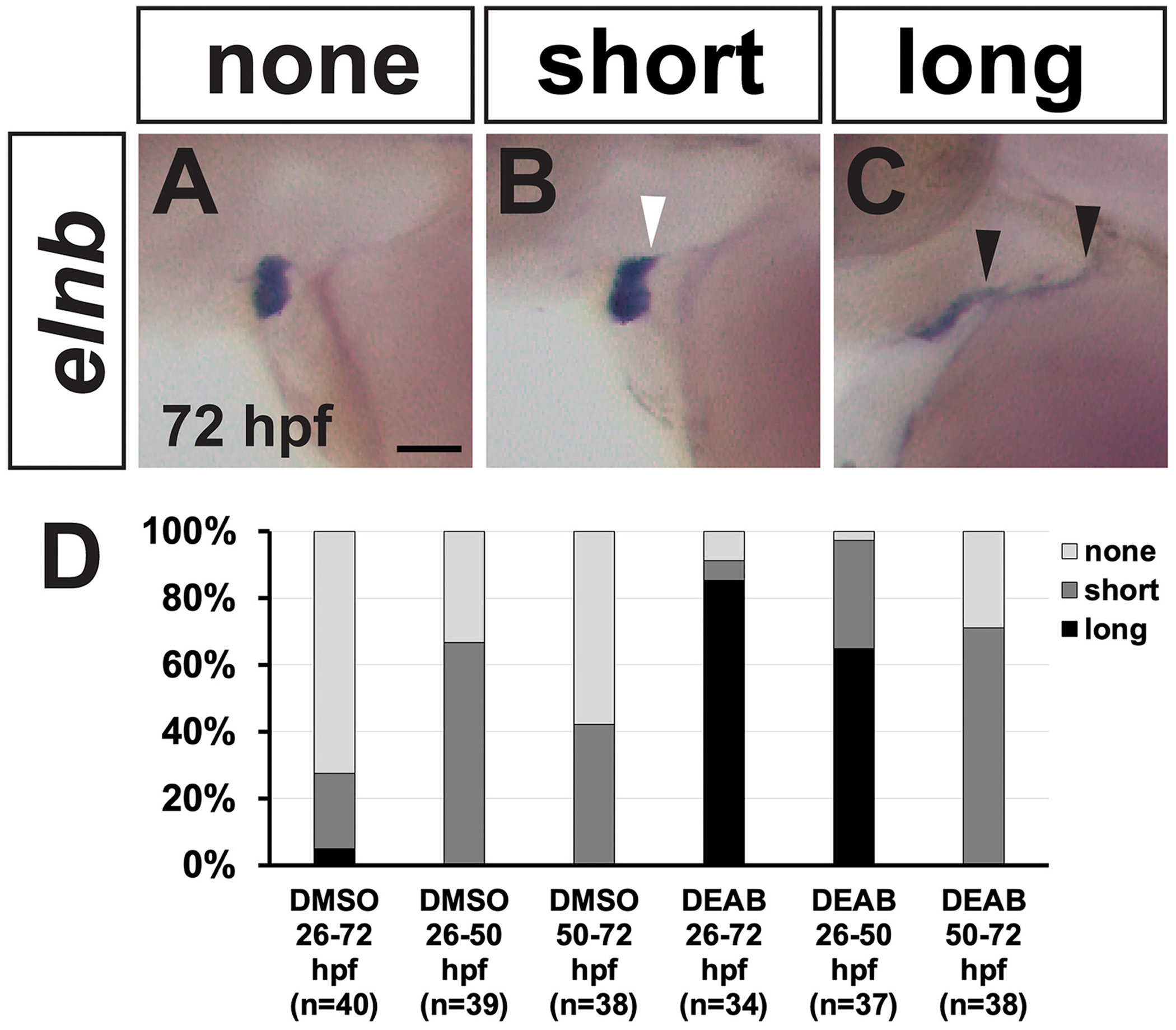
RA acts between 26 hpf and 50 hpf to pattern OFT smooth muscle. (A–C) Images of *elnb* ISH representing none, short, and long categories at 72 hpf. White arrow indicates small posterior *elnb* extension of the “short” category. Black arrowheads indicate length of expanded *elnb* posterior expression of the “long” category. Anterior is to the left. Scale bar is 50 μM. (D) Quantification of phenotype categories for embryos treated with DMSO or DEAB for either 26 hpf – 72 hpf, 26 hpf – 50 hpf, or 50 hpf – 72 hpf.

As there are multiple waves of smooth muscle addition to the OFT from different sources (Cavanaugh et al., 2015; Felker et al., 2018), we wanted to identify the temporal window of RA signaling for OFT smooth muscle patterning. We treated embryos with DEAB from 26 hpf to 50 hpf and from 50 hpf to 72 hpf, then examined *elnb* expression at 72 hpf via ISH. Embryos were placed into three categories based on the *elnb* expression pattern: the “none” category was defined as no visible posterior expression domain (Fig. 10A), the “short” category was defined as a small posterior expression domain (Fig. 10B), and the “long” category was defined as dramatic expansion of the posterior expression domain (Fig. 10C), comparable to the expansion observed in embryos treated with DEAB from 26 hpf to 72 hpf. The “long” category was observed in the 26 hpf to 50 hpf treatment group (Fig. 10D) but not in treatments starting at 50 hpf. Thus, our results are consistent with RA signaling acting between 26 hpf and 50 hpf to direct the distribution of smooth muscle cells between the BA and the posterior ventral aorta.

## 3. Discussion

This study provides insights into the roles of endogenous RA signaling in ventricle and OFT formation specifically during SHF addition to the heart. Our results are consistent with a model in which endogenous RA signaling promotes SHF cardiomyocyte addition to the arterial pole of the heart. Ventricles in late RA-deficient embryos had fewer cardiomyocytes, and our temporal differentiation assay revealed that fewer cells were added to the ventricle by 72 hpf, consistent with reduced SHF addition to the arterial pole. This does not appear to be a result of a reduced SHF progenitor pool or apoptotic cell death, as *ltbp3* expression in the SHF and activated Caspase-3 were unchanged following late RA inhibition. Our EdU results suggest that late RA inhibition does not lead to a significant reduction in cardiomyocyte pro-liferation. EdU pulses were performed prior to the DEAB treatments to allow for continuous DEAB treatment that more closely matched our other experiments, rather than pausing the DEAB treatments to treat with EdU. However, this approach only labels cardiomyocytes that were proliferating prior to the onset of DEAB treatment, and therefore does not address whether initiation of cardiomyocyte proliferation is affected following the application of DEAB. Therefore, we cannot completely rule out the possibility that initiation of cardiomyocyte proliferation is reduced at specific time points following the start of DEAB treatment. Although the SHF progenitor field was unchanged at 33 hpf, we also cannot rule out the possibility that SHF progenitor proliferation is reduced after 33 hpf, as our EdU analysis did not specifically examine the SHF progenitor pool. Lastly, an early wave of cardiac neural crest cells contributes to the ventricular myocardium between 24 hpf and 30 hpf (Cavanaugh et al., 2015), which overlaps with the start of our DEAB treatments. We cannot rule out the possibility that our treatment conditions also affect cardiac neural crest contribution to the ventricle. Overall, our results are consistent with, and extend upon, the mouse studies using conditional RA receptor knockouts that reduce RA signaling during the time of SHF addition, which also results in OFT defects (Li et al., 2010).

There are other mechanisms that may be contributing to the reduced number of ventricular cardiomyocytes. It is possible that SHF progenitors in late RA-deficient embryos are undergoing a fate transformation and are redistributed elsewhere. SHF addition is continuous, with cells first contributing to the arterial pole of the ventricle, and subsequently contributing to the smooth muscle of the BA (Felker et al., 2018). However, we also see a reduction in the BA smooth muscle cell population in late RA-deficient embryos, so it is unlikely that future ventricular cardiomyocytes are directed towards a BA smooth muscle fate. Some cells may be directed toward the ventral aorta endothelium, as we do see a small but significant increase in the overall number of endothelial cells in the ventral aorta. The *nkx2.*5+ cell populations that contribute to the SHF also contribute endothelial cells to the PAAs (Paffett-Lugassy et al., 2017). In Cyp26-deficient embryos, which have an increase in endogenous RA, endothelial cell number in PAAs 3 and 4 is increased, while ventricular cardiomyocyte number is decreased, indicating that RA can influence distribution of cells between the PAAs and the ventricle (Rydeen and Waxman, 2016). Therefore, it is possible that some cells are directed to the pharyngeal arch arteries instead of the ventricular myocardium in late RA-deficient embryos, although we have not quantified this. It is also possible that some SHF cells are directed towards forming smooth muscle to surround the elongated posterior ventral aorta.

The ventricular defects we report seem overtly similar to the teratogenic effects of elevated RA levels during the same time period (Rydeen and Waxman, 2016). However, the mechanisms appear to be different. Teratogenic levels of RA during SHF addition led to extrusion of cardiomyocytes from the ventricle, disorganized cardiomyocyte cell polarity, and increased expression of *mmp9*, indicating that excess RA disrupts overall myocardial integrity. Additionally, elevated RA levels resulted in an enlarged SHF progenitor domain. However, the more laterally-positioned SHF cells had a reduced ability to migrate and integrate into the OFT and instead contributed to the PAAs, further reducing ventricular cardiomyocyte cell number. In contrast, our study explores the requirement for endogenous RA signaling during SHF addition to the OFT, which is not necessarily the opposite of excess RA signaling (D’Aniello and Waxman, 2015). Our experiments did not reveal a change in the SHF progenitor domain size, nor did we observe extruding ventricular cardiomyocytes in late RA-deficient embryos. Thus, it is likely that the reduction in ventricular cardiomyocyte number that we observe arises by a different mechanism than excess RA signaling at similar stages, perhaps by primarily redirecting SHF progenitors to alternative fates, as discussed above. Thus, the two studies are not in conflict, but rather highlight the need for proper levels of RA signaling during SHF addition to the OFT.

Ventral aorta development in zebrafish has not been well characterized. Morphogenesis of the anterior branch of the ventral aorta is driven in part by endothelial proliferation within the BA and subsequent anterior displacement into the ventral aorta (Boezio et al., 2020). TGFβ signaling is involved in both of these processes, by restricting BA endothelial cell proliferation and promoting anterior cell displacement via an unknown mechanism (Abrial et al., 2022; Boezio et al., 2020). Regarding the posterior segment, mutation in the myosin motor domain chaperone *unc45a* leads to lack of ventral aorta fusion posterior to PAA 4 (Anderson et al., 2008). Our results support a role for RA signaling in patterning the ventral aorta by promoting expansion of the anterior segment and restricting expansion of the posterior segment. To our knowledge, this is the first report that implicates any developmental signaling pathway in anterior-posterior patterning of the ventral aorta in zebrafish. It is not known at this time whether the anterior and posterior segments of the ventral aorta have distinct molecular signatures, or if the anterior-posterior patterning simply represents a morphogenic process that occurs differently in the anterior compared to the posterior. Given that RA signaling also plays a key role in aortic arch development in mammals (Anderson and Bamforth, 2022; Hiruma et al., 2002), our results are consistent with conservation of that role in zebrafish.

RA signaling was previously shown to regulate caudal PAA development, as well as patterning of the adjacent pharyngeal endodermal pouches. In zebrafish, inhibiting RA signaling after gastrulation resulted in reduction or loss of caudal pharyngeal endoderm pouches (Kopinke et al., 2006). Likewise, rodent models in which RA signaling is inhibited, including genetic mutants, vitamin A deficiency, and treatment with RA signaling inhibitors, have defects in pharyngeal endoderm pouch morphogenesis, as well as PAAs (Li et al., 2012; Niederreither et al., 2003; Vermot et al., 2003; Wendling et al., 2000; Wilson and Warkany, 1949). It is possible that the defects we see in ventral aorta patterning and PAA spacing in late RA-deficient embryos are secondary to pharyngeal endodermal pouch defects. However, mesodermal-specific knockout of *RARa1* and *RXRa* in mice resulted in reduction or loss of caudal PAAs, while retaining appropriate endoderm pouch morphology, suggesting that RA can act specifically within the mesodermal core of the pharyngeal pouches to pattern the vasculature (Li et al., 2012). The same study also found that in endoderm-specific RAR mutants, some individuals had normal PAA patterning despite lacking endodermal pouch 4, suggesting that PAA patterning does not completely depend on the adjacent endoderm pouches. Thus, patterning of these two adjacent tissues can be separated, so the vascular defects we observe could alternatively be primary defects.

Our results suggest that RA patterns the smooth muscle of the OFT by partitioning smooth muscle cells between the BA and posterior segment of the ventral aorta. Interestingly, the total number of smooth muscle cells surrounding the BA and adjacent posterior branch of the ventral aorta was unchanged in late RA-deficient embryos, but the position of the cells was shifted towards surrounding the dramatically longer posterior ventral aorta segment. The population of smooth muscle cells surrounding the BA is derived from both the SHF and cardiac neural crest (Cavanaugh et al., 2015; Hami et al., 2011). Mature smooth muscle cells are first detected in the BA at 48 hpf using the fluorescent nitric oxide indicator DAF-2DA (Grimes et al., 2006). Neural crest contribution to smooth muscle starts at 50 hpf (Cavanaugh et al., 2015). However, our pulsed DEAB treatment results revealed that RA signaling acts to pattern the OFT smooth muscle distribution earlier, between 26 hpf and 50 hpf. Notably, *tbx1* reporter-expressing cells are first detected at the arterial pole at 39 hpf, and these cells eventually contribute to the BA smooth muscle (Felker et al., 2018). Therefore, RA signaling activity likely affects the behavior of these early BA progenitors, rather than the neural crest cells. Similar to our late RA inhibition experiments, inhibition of FGF signaling from 26 hpf to 34 hpf leads to a smaller BA (Felker et al., 2018), although posterior expansion of the smooth muscle was not noted in that context. Thus, it is likely that the RA and FGF pathways are acting together to pattern the BA smooth muscle.

The source and timing of the smooth muscle cells surrounding the posterior ventral aorta branch has not been specifically investigated. The shift in distribution of smooth muscle cells in late RA-deficient embryos may be secondary to the change in ventral aorta segment length. One possibility is that smooth muscle cells first surround the posterior ventral aorta segment, and then contribute to the BA. In wild type embryos, the contribution to the posterior smooth muscle region would be very small, due to the short posterior ventral aorta segment. It is possible that in late RA-deficient embryos, a greater number of smooth muscle cells are required to surround the elongated posterior ventral aorta, leaving an insufficient number remaining for BA contribution. At the time point of our smooth muscle analyses, smooth muscle cells are accumulating around the anterior branch of the ventral aorta, yet we did not observe significant Elnb protein expression surrounding the anterior branch. *elnb* mRNA expression is not reported in the area of the anterior ventral aorta through 5 dpf (Miao et al., 2007), but other markers of smooth muscle such as *tagln* and *acta2* are expressed (Santoro et al., 2009). It is possible that *elnb* is expressed in low levels that are not detected by ISH in the anterior region. Alternatively, it is possible that smooth muscle cells surrounding the anterior and posterior regions of the ventral aorta have different transcriptional profiles.

## 4. Conclusions

Altogether, we have shown that inhibiting RA signaling during the time period of SHF addition in zebrafish results in smaller ventricles with reduced cardiomyocyte number, due in part to reduced addition of SHF-derived cardiomyocytes. Moreover, we showed that late RA inhibition results in shifted smooth muscle distribution within the OFT and ventral aorta, along with a lengthening of the posterior ventral aorta segment and a failure of the junction between the BA and the ventral aorta to rearrange properly. Our results provide new insight into the role of RA signaling in promoting SHF addition to the arterial pole of the ventricle and have uncovered a novel role for RA signaling in anterior-posterior patterning of the ventral aorta. Given that RA signaling is crucial for aortic arch development in rodent models, our results in zebrafish are consistent with a conserved role for RA signaling in aorta development in vertebrates.

## 5. Materials and methods

### 5.1. Ethics statement

All zebrafish husbandry and experiments were performed using standard protocols as approved by the Miami University IACUC.

### 5.2. Zebrafish lines

We used the following zebrafish lines for this study: AB wild type, *Tg (myl7:NLS-KikGR*) (Lazic and Scott, 2011)*, Tg(kdrl:nlsEGFP)* (Blum et al., 2008).

### 5.3. DEAB treatments

DEAB treatments were performed as previously described (Waxman et al., 2008). 20 embryos were placed into each glass vial, transfer liquid was removed and replaced with 2 mL of embryo media containing 1-phenyl-2-thiourea (PTU) to prevent pigment accumulation. For control treatments, 2 μL of DMSO was added to each vial. For experimental treatments, 2.5 μL of DEAB (Sigma) was added to each vial, for a final concentration of 12.5 μM. Vials were gently mixed and caps placed lightly on the tubes to allow for air flow. Embryos were incubated at 28.5 °C on a nutator in the dark until the desired end point. All treatments began at 26 hpf and ended at 72 hpf unless otherwise noted. For washout treatments, DEAB was removed at 50 hpf and embryos were washed at least three times with an excess of embryo media, then allowed to develop in the glass vials until the desired end point.

### 5.4. In situ hybridization

In situ hybridization (ISH) was performed as previously described (Oxtoby and Jowett, 1993). Probes were used for the following genes: *amhc/myh6* (ZDB-GENE-031112-1), *cyp26a1* (ZDB-GENE-990415-44), *elnb* (ZDB-GENE-061212-2), *ltbp3* (ZDB-GENE-060526-130), *myl7* (ZDB-GENE-991019-3), *vmhc/myh7* (ZDB-GENE-991123-5). Following ISH, embryos were transferred to 80% glycerol for imaging using either a Zeiss Discovery V8 or an Olympus SZX-12 stereomicroscope.

### 5.5. Immunohistochemistry, cell counting, and vessel measurements

Immunohistochemistry (IHC) for cell counting and ventral aorta analyses was performed as previously described (Waxman et al., 2008). Embryos were fixed in 1% paraformaldehyde in PBS for 1 h at room temperature, followed by a wash with 1x PBS and two washes in 0.2% saponin/1x PBS. Embryos were blocked for 1 h at room temperature in saponin blocking solution (0.2% saponin, 10% sheep serum, 1x PBS, 2 mg/mL BSA), then incubated in primary antibodies diluted in saponin blocking solution overnight at 4° C. Embryos were washed three times for 10 min each in 0.2% saponin/1x PBS, then incubated in secondary antibodies diluted in saponin blocking solution for either 2 h at room temperature or overnight at 4°C. Embryos were washed three times for 10 min each in 0.2% saponin/1x PBS, then transferred to 0.1% saponin/1x PBS before imaging. The following antibodies were used: chicken anti-GFP (1:1000, Abcam), rabbit anti-Elnb (1:100) (Holowiecki et al., 2020; Song et al., 2019), anti-chicken Alexa 488, anti-rabbit Alexa 488, anti-rabbit Alexa 555. Eln2-processed embryos were also stained for DAPI to mark cell nuclei. IHC experiments were imaged in 0.1% saponin using a Zeiss LSM 710 inverted confocal microscope. Cell counting from images was performed using Fiji (NIH) as previously described (Waxman et al., 2008). Because Elnb cell counting utilized DAPI to mark cell nuclei, cells were counted using the Z-stack to exclude the endothelial cell nuclei lining the BA and ventral aorta, which are ensheathed by the Elnb-expressing smooth muscle cells. Only nuclei that were surrounded by Elnb expression were counted as smooth muscle cells. Student’s *t*-test was used to determine statistical significance.

For ventral aorta measurements, the center of the bulbus arteriosus was determined as the boundary between the anterior and posterior segments. Each segment was measured separately using Fiji, then total length was calculated. In cases where the anterior ventral aorta was split on the left and right sides, but had partial fusion at a distance from the BA, the distance from the center of the BA to the point of fusion was measured on both the left and right sides, and the average of the two lengths was calculated. The center of the BA was also used as the reference point to measure the distance of the OFT to PAA 4. Student’s *t*-test was used to determine statistical significance.

IHC for apoptotic cells using the rabbit anti-activated Caspase-3 antibody (1:250, BD Biosciences) was performed as previously described (Sorrells et al., 2013). 32 hpf embryos were co-stained with the mouse MF-20 antibody (1:10, Developmental Studies Hybridoma Bank) to label the whole heart.

### 5.6. Temporal differentiation assay

Temporal differentiation assays were performed using the *myl7:NLS-KikGR* similar to what is previously described (Rydeen and Waxman, 2016). Embryos were sorted at 26 hpf for the presence of the transgene. Photoconversion was performed using an Olympus AX-70 microscope. Embryos were exposed to light through a DAPI filter for 1 min. Full photoconversion was confirmed in both green and red channels for each embryo. Photoconverted embryos were then immediately treated with DMSO or DEAB as described above. For endpoint analysis, embryos were fixed for 1 h in 1% paraformaldehyde in PBS at room temperature, followed by a wash with 1x PBS and two washes in 0.2% saponin/1x PBS. Embryos were imaged on the same day in 0.2% saponin using a Zeiss LSM 710 confocal microscope. Green-only cells were counted from acquired images using Fiji. Student’s *t*-test was used to determine statistical significance.

### 5.7. EdU labeling

EdU labeling was performed using the Click-iT EdU Alexa Fluor 594 Imaging Kit (Life Technologies) similar to previous reports (Holowiecki et al., 2020; Martin et al., 2023). Embryos carrying the *myl7:NLS-KikGR* transgene were dechorionated and placed into glass vials at 26 hpf, with 20 embryos per vial. They were incubated in 10 mM EdU for 20 min on ice, then washed several times with embryo media before being placed into DMSO or DEAB treatments as described above. At 72 hpf, embryos were fixed in 1% paraformaldehyde in PBS for 1 h at room temperature, followed by a wash with 1x PBS and two washes in 0.2% saponin/1x PBS. The Click-iT reaction was then performed following the manufacturer’s protocol. Embryos were imaged on the same day in 0.2% saponin using a Zeiss LSM 710 confocal microscope. The proliferation index was calculated as the number of EdU+/*NLS-KikGR* + ventricular cardiomyocytes divided by the total number of *NLS-KikGR* + ventricular cardiomyocytes.

## Data Availability

Data will be made available on request.
